# Digital Solutions in HF Education: What Can Patients and Clinicians Gain?

**DOI:** 10.1007/s11897-025-00738-5

**Published:** 2026-01-09

**Authors:** Teresa Castiello, Loreena Hill, Sharon Man, Sheref Elseidy, Daniel Griffin, Mark J. Schuuring

**Affiliations:** 1 Present Address: Cardiology Department, Royal Free Hospital, MIAL Healthcare, London, United Kingdom; 2 School of Nursing & Paramedic Science, Ulster University, Northern Ireland, United Kingdom; 3 Department of Cardiovascular Sciences, University of Leicester, University Hospitals Plymouth NHS Trust, Leicester, United Kingdom; 4 Department of cardiology, Ain Shams University hospitals, Cairo, Egypt; 5 Guy’s and St Thomas’ NHS Foundation Trust, London, United Kingdom; 6 Department of Biomedical Signals and Systems, University of Twente, Enschede, The Netherlands; 7 Department of Cardiology, Medisch Spectrum Twente, Enschede, the Netherlands

**Keywords:** Digital education, Heart failure, Patient education, Self-management, E-health, Artificial intelligence

## Abstract

**Purpose of Review:**

Heart failure (HF) imposes an expanding global health burden, necessitating innovative approaches to education for both patients and clinicians. This review evaluates the evolving landscape of digital health tools in HF education and examines how these technologies may enhance accessibility, personalisation, and engagement in contemporary care.

**Recent Findings:**

Emerging evidence demonstrates that digital solutions—ranging from remote educational platforms and interactive applications to AI-assisted learning and immersive reality technologies—can meaningfully improve patient self-management, support clinician knowledge acquisition, and strengthen overall care quality. These tools offer substantial advantages, including remote access to high-quality information, dynamic and interactive learning experiences, and opportunities for continuous monitoring. Nonetheless, challenges persist, particularly regarding equitable access, digital literacy, data quality, and integration into existing clinical workflows.

**Summary:**

Digital technologies hold considerable promise in optimising HF education for both patients and clinicians. When effectively implemented, they have the potential to improve patient outcomes, enhance clinical decision-making, and support more efficient healthcare delivery. Continued innovation—particularly in AI, virtual and augmented reality, and personalised learning systems—will be essential to address remaining limitations and fully realise the transformative potential of digital HF education.

**Graphical Abstract:**

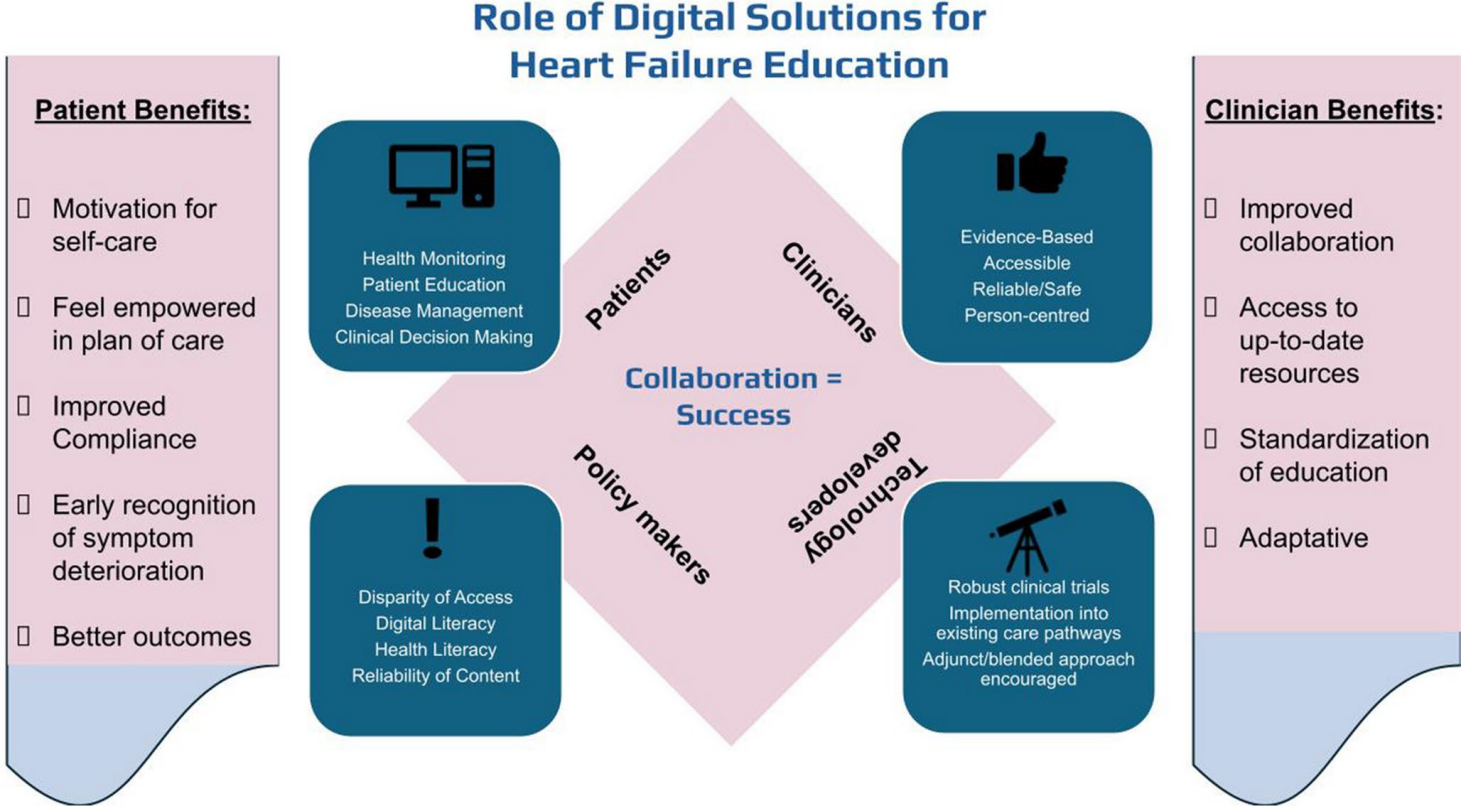

## Introduction

Heart failure (HF) represents a significant and growing global health challenge, affecting an estimated 64 million individuals worldwide with a steadily increasing prevalence driven by an aging population and a rising burden of comorbidities [1, 2]. The escalating demand for traditional healthcare services necessitates the integration of innovative technologies into the medical pathway and the core curriculum for clinicians.

The World Health Organisation (WHO) defined digital health as “the integration of digital technologies into healthcare systems to enhance the efficiency of healthcare delivery, improve patient outcomes, and empower individuals to manage their health more effectively using digital solutions and platforms” [3]. This definition can encompass a broad spectrum of applications, including health monitoring, disease management, patient education, and clinical decision-making. Artificial intelligence (AI), a field of computer science focused on creating systems capable of learning, reasoning, and acting autonomously [4], is increasingly being integrated into modern-day digital health solutions.

Education is fundamental to empowering both patients and clinicians in the effective recognition and management of HF. For patients, comprehensive education fosters a deeper understanding of their illness, facilitating symptom identification, promoting adherence to treatment plans and lifestyle modifications, and encouraging patients to practice proactive self-care. Digital solutions offer diverse avenues for delivering patient education, ranging from established remote platforms to cutting-edge AI-driven simulations and Virtual Reality (VR) training sessions [5]. Similarly, clinicians benefit from readily accessible educational resources, evidence-based guidelines, and updates on diagnostic and therapeutic strategies to ensure timely and high-quality patient care.

This review aims to explore the current landscape of digital solutions in HF education for both patients and clinicians. We will discuss the potential benefits these technologies offer, the challenges and limitations associated with their implementation, and future perspectives on how digital health can be effectively leveraged to enhance knowledge, improve engagement, and ultimately optimise outcomes in the management of patients with heart failure.

## Defining Digital Solutions in Healthcare Education

Digital solutions in healthcare education encompass a wide array of technologies designed to deliver, enhance, and support learning for both patients and clinicians. These solutions leverage digital platforms and tools to provide accessible, interactive, and often personalised educational experiences. Key components include:

- **E-learning Platforms**: Online platforms offering structured educational modules, videos, interactive simulations, and assessment tools.
- **Mobile Health Applications (mHealth)**: Apps for smartphones and tablets providing patient education, medication reminders, symptom tracking, and communication with the healthcare team.
- **Telehealth and Virtual Conferencing**: Platforms enabling remote lectures, webinars, and virtual meetings for clinicians for professional development, as well as remote patient consultations.
- **Augmented Reality (AR) and Virtual Reality (VR)**: Immersive technologies creating interactive learning environments for visualising complex concepts and simulating clinical scenarios.
- **Artificial Intelligence (AI)**: AI-powered tools for personalised learning pathways, automated feedback, and simulated patient interactions.

These digital solutions, when applied across various fields in healthcare education, can provide:

- **Knowledge Acquisition**: Providing access to up-to-date medical information, guidelines, and research findings.
- **Skill Development**: Utilising simulations and interactive platforms to enhance clinical skills and decision-making.
- **Patient Engagement and Activation**: Empowering patients with knowledge and tools for self-management and adherence to treatment.
- **Continuing Professional Development (CPD)**: Offers flexible and accessible learning opportunities for clinicians to maintain and enhance their expertise.

## Education for Clinicians

**Fig. 1:**
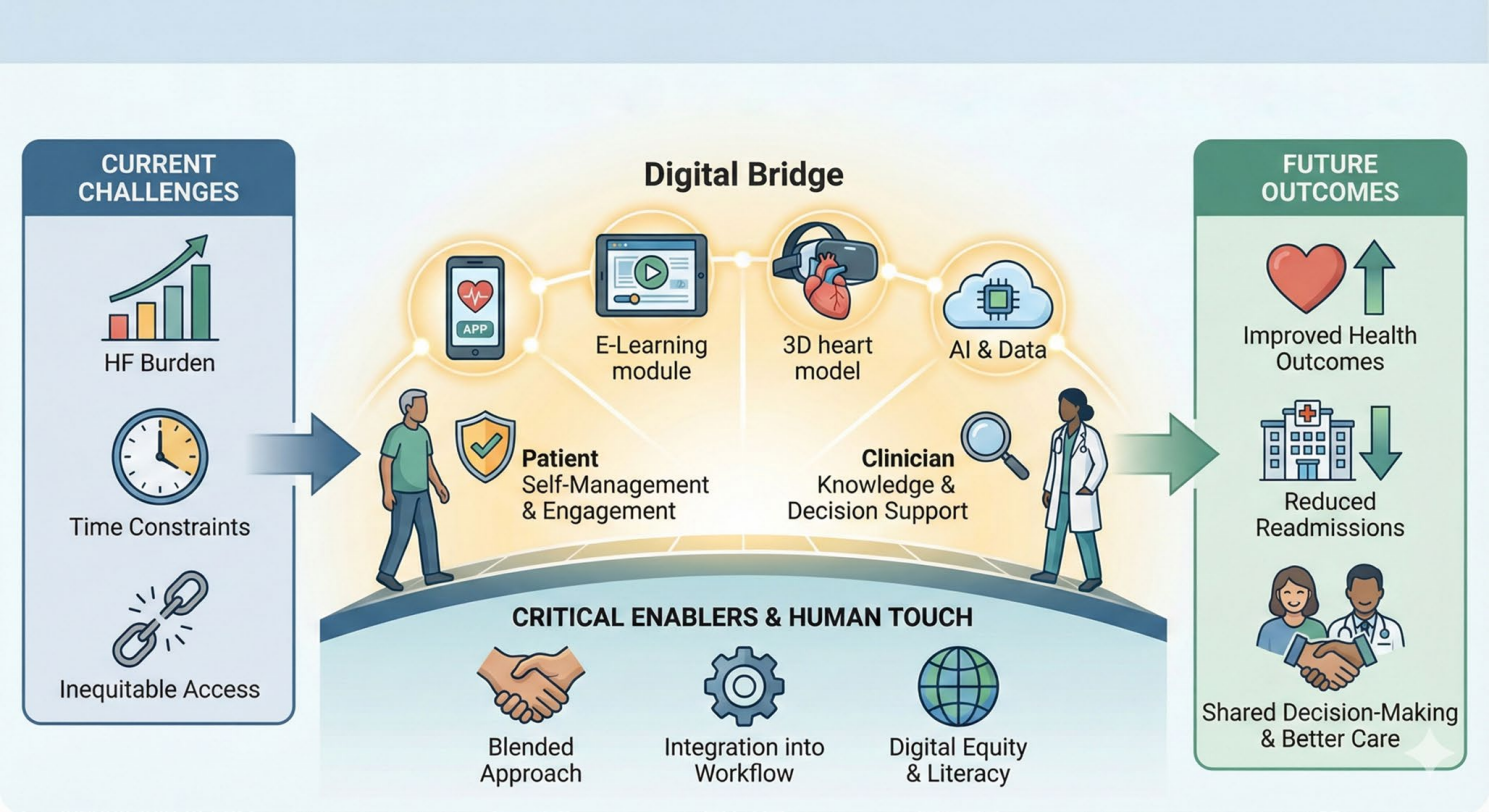
Infographic summary. Digital Solutions in heart failure education: bridging gaps and empowering care

Digital solutions offer a multitude of potential benefits for the education of clinicians in heart failure management. These include enhanced accessibility to current information, interactive learning modalities, opportunities for remote collaboration, personalised training programs, support for continuing professional development, and the potential for standardising educational levels, particularly in underserved regions Fig. 1.

## Benefits of Digital Solutions for Clinician Education

- **Accessibility to Up-to-Date Information**: Digital platforms transcend geographical limitations, providing clinicians with seamless access to a vast repository of resources, including medical journals, international guidelines from organisations like the European Society of Cardiology (ESC) and the Heart Failure Association (HFA), and up-to-date empirical studies. This equitable dissemination of knowledge helps to bridge gaps in expertise across different regions and ensures clinicians have the latest information regardless of their location [6]. The ESC chat AI, is an AI-powered tool designed to deliver immediate, citation-backed responses to clinical queries by synthesizing information exclusively from current ESC Clinical Practice Guidelines. This tool expedites the retrieval of cross-disciplinary insights, allowing clinicians to rapidly access relevant guidance for complex scenarios like multimorbidity by synthesizing recommendations from the entire library of ESC guidelines rather than relying solely on the specific Heart Failure document.
- **Interactive and Personalised Learning**: Digital solutions can cater to diverse learning styles through formats such as text, videos, visual explainers, and interactive simulations, allowing healthcare professionals to focus on the HF-related competencies they most need to develop. In heart failure care, this may include modules on optimising guideline-directed medical therapy, interpreting congestion markers, or managing device-treated patients. Some platforms also facilitate the exchange of HF-specific expertise, fostering discussion forums and virtual communities centred on HF management. These collaborative environments promote continuous learning and peer-to-peer support, which are particularly valuable given the rapid evolution of HF therapeutics and technologies. Mobile applications such as MedShr [7] have demonstrated the potential of digital platforms to enhance patient care by enabling global clinical case discussions, including complex HF presentations requiring multidisciplinary input.
- **Effectiveness of Digital Learning Modalities**: While debates persist regarding the superiority of online versus in-person learning, existing evidence suggests that technology-enhanced simulation training significantly improves clinicians’ knowledge, skills, and behaviour, with moderate effects on patient outcomes [8]. These findings are highly relevant to HF, where effective management often depends on accurate clinical assessment, timely therapeutic adjustments, and multidisciplinary coordination—skills that can be practised and refined through simulation-based digital tools. Blended learning approaches, combining online modules with face-to-face instruction, are comparable or even superior to traditional methods in training practising clinicians [9, 10]. Despite these encouraging results, the heterogeneity of existing studies highlights the need for randomised controlled trials specifically designed to evaluate digital education modalities within HF care. Such trials could clarify the extent to which digital learning enhances HF-specific competencies, influences patient trajectories, and supports the adoption of complex therapies.

## A New Era of Digitalisation in Heart Failure Clinician Education

The traditional model of in-person and self-directed learning for HF clinicians is being rapidly augmented—and in some cases reshaped—by digital technologies across all levels of training. Virtual conferencing platforms such as Microsoft Teams have become integral to HF education, enabling both live and on-demand dissemination of guideline updates, case-based discussions, and expert-led seminars to geographically diverse audiences. Organisations such as the British Society for Heart Failure and the ESC have effectively used webinars to share contemporary insights into HF diagnostics, pharmacotherapy, and device management, thereby democratising access to specialist knowledge for HF clinicians as well as physicians in primary care, emergency medicine, general cardiology, and other disciplines who manage HF patients [11–13].

However, this ease of access is accompanied by notable challenges. Clinicians may encounter digital educational material of inconsistent or unverified quality—an issue particularly concerning in HF, where rapidly evolving evidence demands accuracy. Videos on publicly accessible platforms such as YouTube often lack peer review and may inadvertently convey outdated or non–evidence-based information [14]. Additionally, “Zoom fatigue,” characterised by reduced concentration during prolonged virtual sessions, has been widely reported and can hinder the assimilation of complex HF concepts such as haemodynamic interpretation or guideline-directed medical therapy optimisation [11, 15, 16].

E-learning modules are now well-established in cardiology training [17, 18]. Comparative studies have shown that e-learning can improve early retention of clinical information [19] and that blended learning approaches enhance performance in skills such as ECG interpretation [10]—competencies highly relevant to HF clinicians and non-HF specialists alike. Nonetheless, despite these positive findings, there remains a notable lack of research specifically evaluating e-learning platforms designed for HF education, highlighting a gap in evidence for this population.

**Fig. 2:**
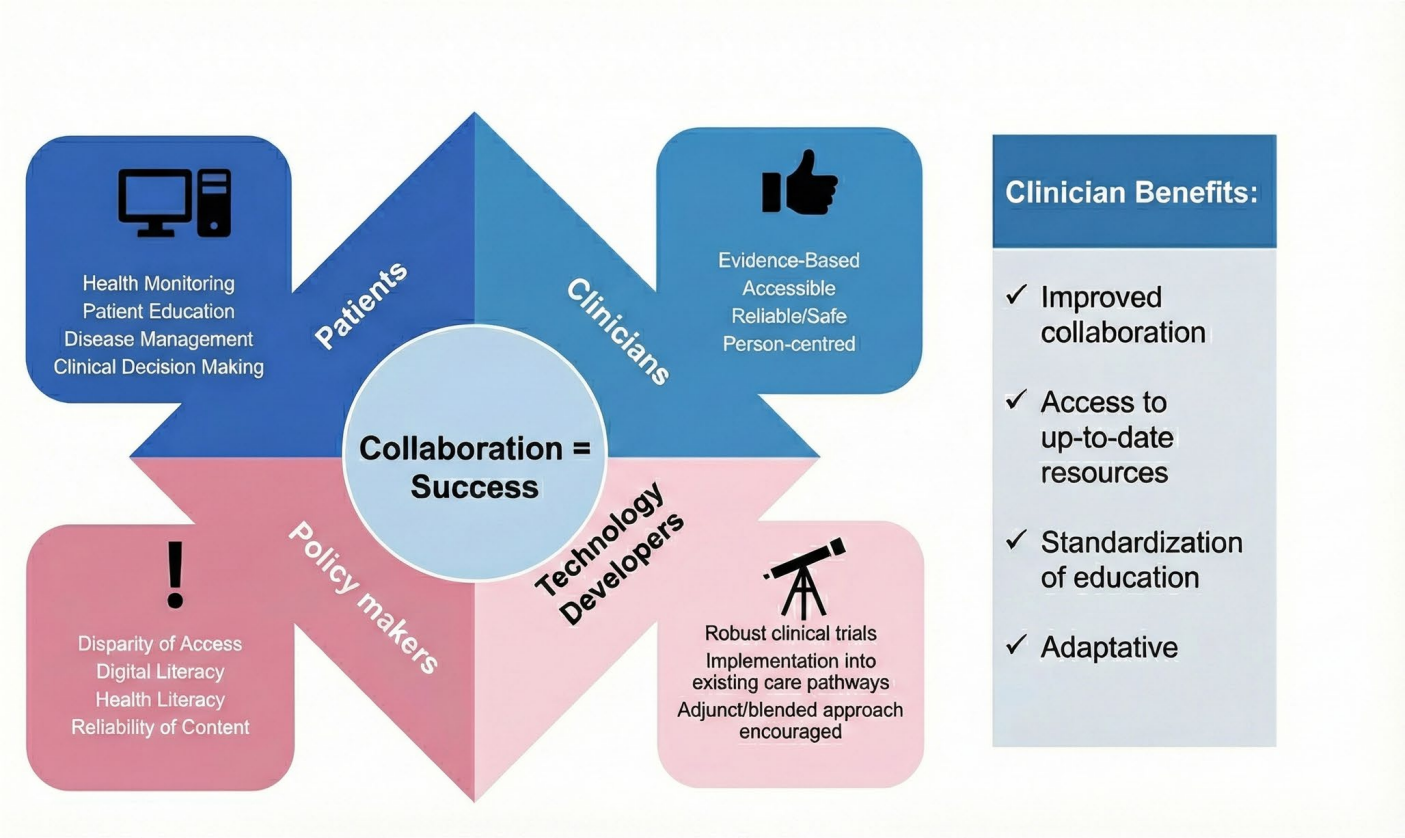
Rold of digital solutions for heart failure education

Advances in Augmented Reality (AR) and Virtual Reality (VR) are also introducing innovative opportunities for HF training. These immersive tools allow clinicians to visualise complex cardiac structures, ventricular remodelling, valvular pathology, and device–heart interactions—elements central to HF pathophysiology—in dynamic three-dimensional formats [20]. Meta-analyses show moderate improvements in post-intervention test scores using VR to teach anatomy [21], suggesting a promising future for AR/VR-enhanced HF education. Further research is needed to assess the specificity, scalability, and real-world impact of these modalities within HF training programmes Fig. 2.

## Future Perspectives on Implementing Digital Health for Heart Failure Clinician Education

**Fig. 3:**
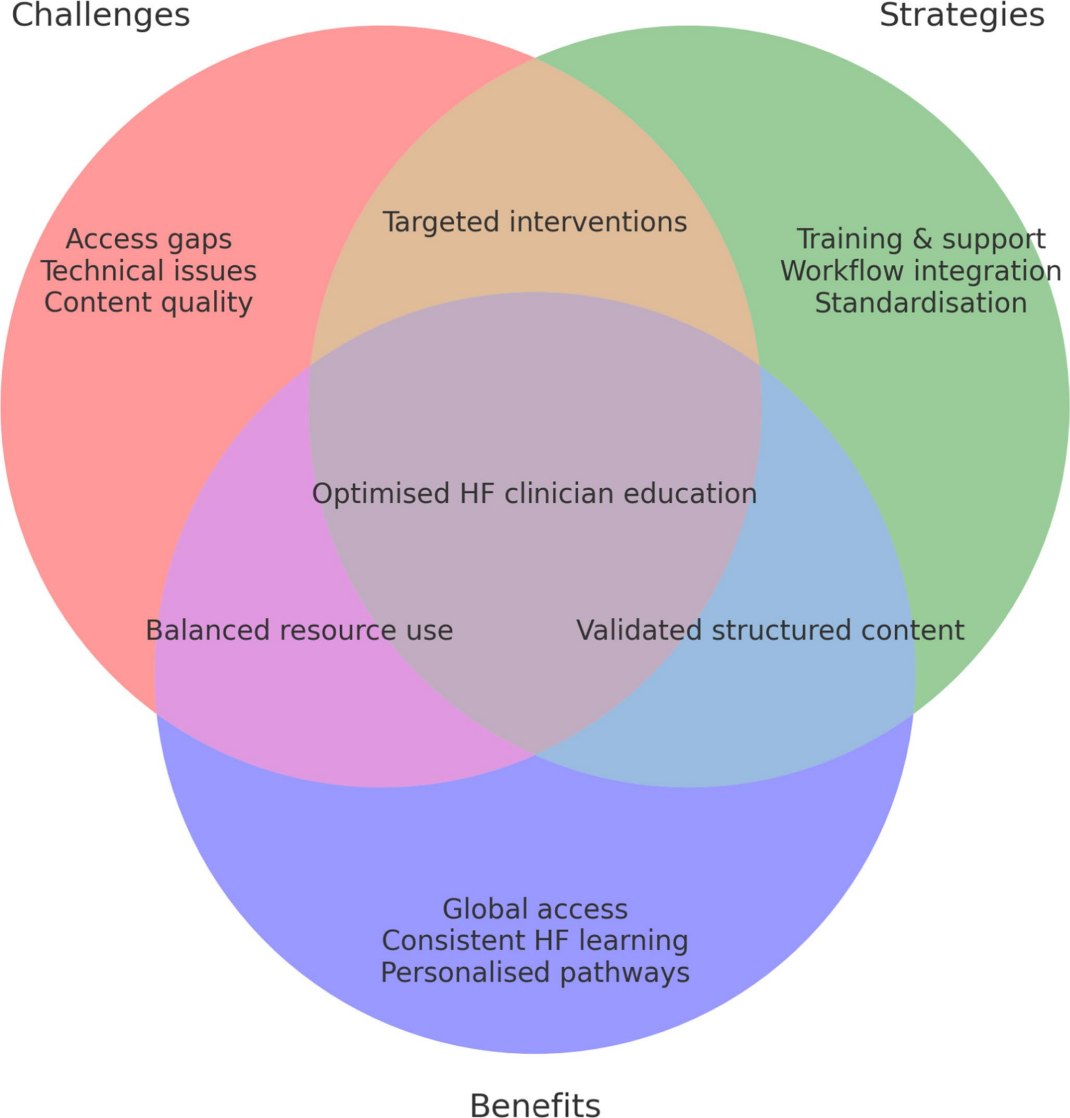
Digital solutions in HF clinician education

The continued evolution of digital technologies may lead to an increasingly blended approach to delivering HF education for clinicians, through the integration of a range of digital interventions to complement and, in some cases, replace traditional teaching methods. Augmented Reality (AR) and Virtual Reality (VR) have the potential to offer novel and effective ways to teach the fundamental aspects of anatomy and pathophysiology, leading to improved comprehension in clinical learners Fig 3.

The development of metaverse technology [22], a virtual universe accessible through AR/VR, represents a potential revolution in medical and HF education. Trainees could attend immersive 3D virtual lectures and seminars delivered by international experts. Early examples, such as lectures in the metaverse offered at Queen Mary University London [23], suggest a more engaging and interactive experience compared to current virtual conferencing or traditional lectures. Furthermore, clinicians have the opportunity to interact with virtual patients exhibiting various symptoms and clinical signs of HF. Combined with advancements in Artificial Intelligence (AI) [24], these simulated patients could provide comprehensive medical histories, demonstrate dynamic symptoms and allow for the visualisation of internal 3D structures thereby enhancing clinicians’ knowledge on, and the relationship between anatomy, pathophysiology, presentation and management.

This technology, once widely accessible, could overcome geographical barriers to high-quality HF education, making access to technology the primary determinant rather than the clinician’s location. While initial adoption may be concentrated in more affluent regions with better technological infrastructure, the increasing affordability and widespread availability of these tools hold the potential to transform HF education (and medical education more broadly) in underserved areas across the globe (Table 1, [25]).

**Table 1:** Gaps in evidence and future research directions

Area of Investigation	Specific Research Questions
Efficacy of Specific Digital Modalities	What is the comparative effectiveness of different digital education formats (e.g., apps, e-learning modules, VR/AR) on clinician knowledge, skills and patient outcomes in HF?
Integration with Clinical Workflow	How can digital education tools be seamlessly integrated into the daily workflow of HF clinicians to optimise their use and minimise burden?
Personalisation and Tailoring	What are the most effective strategies for personalising digital education content based on individual patient characteristics (e.g., health literacy, cognitive function, preferences) and clinician learning styles?
Addressing the Digital Divide	What interventions can effectively bridge the digital divide and ensure equitable access to digital education resources for all HF patients and clinicians, regardless of socio-economic status or geographical location?
Cost-Effectiveness and Sustainability	What is the long-term cost-effectiveness of different digital education strategies for HF management, considering both direct and indirect costs? What are sustainable models for funding and maintaining these platforms?
Impact on Shared Decision-Making	How do digital education tools influence the process of shared decision-making between patients and clinicians in the context of HF management?
Ethical Implications and Data Security	What are the ethical considerations and best practices for ensuring data privacy, security and responsible use of AI in digital HF education tools?
Role of AI in Personalised Education and Prediction	How can AI be leveraged to create more adaptive and predictive digital education tools that anticipate learning needs and personalise content delivery for both patients and clinicians?
Impact on Clinician Well-being and Burnout	Does the integration of digital education tools impact clinician well-being and burnout rates? What strategies can be implemented to mitigate potential negative effects?

## Challenges and Limitations of Using Digital Solutions for Education of Heart Failure Clinician

Despite the immense potential of digital solutions in education, several challenges and limitations may hinder their widespread and effective application in HF clinician training.

- **Disparities in Access and Digital Literacy**: Unequal access to technology and varying levels of digital literacy among clinicians represent a significant obstacle. As AI becomes increasingly integrated into healthcare, clinicians require ongoing training to effectively utilise these applications, including understanding the basics of AI, data evaluation, workflow integration, bias control, and ethical considerations [26]. A lack of necessary digital skills can lead to unequal educational opportunities, particularly in resource-limited settings.
- **Technical Issues**: Digital platforms are susceptible to technical problems such as glitches, connectivity issues, and device incompatibility, which can disrupt the learning process, frustrate users, and reduce engagement.
- **Quality Control of Information**: Ensuring the quality and reliability of educational content on digital platforms is a major concern due to the potential exposure to unverified or inaccurate sources. Reviews of digital resources in undergraduate education have revealed a significant focus on satisfaction levels rather than deeper learning outcomes [27]. Establishing stringent quality standards by cardiology societies to identify reliable, peer-reviewed educational resources is crucial.
- **Lack of Personalisation and Real-Time Interaction**: While some digital solutions offer personalised learning, many still employ a generalised approach that may not cater to the unique needs and learning styles of individual clinicians. Furthermore, the often-limited opportunities for real-time interaction with instructors or peers can hinder the development of critical thinking and problem-solving skills through questioning, clarification and feedback.
- **Financial Investment and Sustainability**: The development and maintenance of high-quality digital educational platforms require substantial financial investment in technology, content creation, and ongoing platform upkeep. Ensuring the sustainability of these platforms, particularly in low-resource settings, can be challenging. The cost of subscription and barriers to obtaining reimbursement for digital educational platforms may deter some clinical users from utilising resources that require financial payment.

Addressing these challenges related to access, quality and personalisation is crucial for maximising the effectiveness of digital platforms in healthcare education. Further research is also essential to definitively establish their impact on complex learning outcomes in clinicians and cost-effectiveness in management of HF in all healthcare settings [27].

## Benefits of Using Digital Solutions for Patients’ Education

Educating patients with HF is a fundamental responsibility of the multidisciplinary HF team, aiming to improve treatment adherence, strengthen self-care behaviours, and reduce avoidable hospitalisations [28, 29]. As HF prevalence continues to rise, digital technologies offer valuable opportunities to support patient education and engagement [30]. Various tools—including mobile applications and remote monitoring platforms—have shown promise in motivating patients through accessible interfaces and real-time feedback [31, 32]. A study from India [33] highlighted the breadth of functionalities currently available in HF-related apps, including medication reminders (88.6%), educational content (84.3%), direct messaging with clinicians (84.3%), physical activity tracking (81.4%), symptom monitoring (78.6%), dietary guidance (78.6%), and weight management support (72.9%).

Evidence also suggests that structured digital educational resources can improve patient outcomes. For example, a Dutch randomised controlled trial [34] demonstrated that engagement with the ESC/HFA website *heartfailurematters.org*, which provides multilingual educational videos and patient-friendly explanations, improved self-care and quality of life at 3 months, although these benefits diminished by 12 months. This waning effect underscores the likelihood that digital education alone may not sustain behavioural change over time. It therefore supports the need for periodic personal contact or “booster sessions”, delivered by HF nurses, clinicians, or digital health navigators, to reinforce engagement, maintain motivation, and ensure long-term adherence to digital tools.

Despite their promise, digital solutions also introduce new challenges. Patients may encounter highly variable online information quality, including exposure to misinformation or so-called “fake news,” highlighting the clinicians’ responsibility to actively guide patients toward trustworthy, evidence-based digital resources. Additional barriers include internet availability, limited digital literacy, and reduced engagement—particularly in older or more vulnerable populations. In one study of hospitalised older adults (mean age 81.5 years), 72% were unable to use an HF-specific app, and non-engagement was associated with higher readmission risk [36]. Similarly, a Cochrane review [37] found uncertainty regarding the impact of mHealth-delivered education on quality of life, HF-related hospitalisation, or self-care, largely due to heterogeneity across interventions. Collectively, these findings highlight that digital solutions should complement, rather than replace, traditional educational approaches [38], and that structured clinician support remains essential for ensuring patients access reliable, high-quality information.

## Future Perspectives on Implementing Digital Health for Patients’ Education

Many patients and clinicians recognise the benefits of digital health, a trend accelerated by the COVID-19 pandemic. However, concerns persist regarding the potential loss of the “personal touch” in the patient-clinician relationship [39]. A complementary approach that integrates digital tools while maintaining human interaction can address these concerns and better appeal to patients managing their chronic conditions at home.

Incorporating digital educational platforms can empower patients to access information at their own pace, improving their understanding and motivation for self-care, ultimately fostering shared decision-making for improved clinical outcomes [40, 41]. A key challenge in delivering education via digital platforms is patients’ health literacy, which has been shown to influence uptake and engagement [41]. Personalising the education provided by the device based on the patient’s current health status, cognitive abilities, and psychological needs could be a valuable strategy to enhance self-management. Ultimately, personalised digital education aims to provide the ‘right technology to the right patient at the right time’ [42].

The feasibility of digital cardiac rehabilitation programs has been explored [43], with patients with HF showing greater interest, compared to patients post-coronary bypass surgery. Interestingly, the researchers found that the patient’s attitude toward a healthy lifestyle was a significant predictor of their willingness to participate. Future implementation strategies may need to consider patients’ health literacy, digital literacy and motivation to make the necessary lifestyle choices to ensure positive engagement. Finally, the integration of easily accessible, tailored digital devices with validated AI technology holds significant promise for the future of not just in education but also in HF care delivery (Table 1, [44]).

## Role and Challenges of Digital Solutions in Education of Patients with Heart Failure

The primary goals of educating patients living with HF include improving their understanding of the diagnosis, enabling their recognition of worsening symptoms, informing them about accessing specialist support networks, and explaining how pharmacological and non-pharmacological interventions can alleviate symptoms and improve prognosis. Achieving these learning objectives is expected to enhance patients’ adherence to treatment and their ability to self-monitor their condition [45, 46]. Ultimately, effective patient and clinician education aims to improve patients’ quality of life, reduce symptom burden, decrease hospital admissions for decompensated HF, and lower mortality from major adverse cardiovascular events.

Achieving these educational goals requires implementation or redesign of care pathways, deployment of appropriate tools and equipment, trained health care professionals, and dedicated time for patient and clinician training, coupled with regular audits to evaluate the effectiveness of the education provided. Traditionally, education of patients and caregivers has been delivered through clinician - or nurse-led consultations and clinics. Patients with cardiac implantable devices for HF also receive education by allied professionals (APs) in specialised device clinics.

Recent advancements in HF digital health have been driven by the proliferation of digital technologies, such as mobile health apps and wearable sensors. These tools can continuously monitor a multitude of physiological data, providing real-time feedback to clinicians via remote monitoring platforms. These digital technologies offer opportunities for improved and timely access to specialist treatment with guideline-directed medical therapy (GDMT), potentially preventing deterioration and avoiding hospitalisations [47–49].Crucially, devices intended for clinical application must obtain appropriate regulatory certification, such as CE marking or FDA approval, to ensure technical accuracy, clinical safety, and data integrity. Beyond regulatory clearance, widespread clinical adoption also warrants rigorous quality control, with digital applications undergoing formal validation through randomized controlled trials analogous to those required for pharmaceutical therapies. Furthermore, the clinical utility of remote monitoring technologies depends critically on the successful implementation of a complete “care cycle,” whereby transmitted physiological data consistently trigger timely, appropriate, and clinically meaningful therapeutic actions, rather than passive data accumulation alone [50].

However, for this model to be effective, both patients and clinicians need to be educated on which digital solutions to use, their functionalities and limitations, and how to integrate them effectively into daily management. Currently, a variety of free mobile health apps are available for HF patients [51], such as HF Health Storylines (developed by the Heart Failure Society of America) and HF Helper (developed with the American Heart Association), which allow patients to track medications, symptoms, mood, activity levels, and vital signs, facilitating data sharing with clinicians and providing educational resources and peer support.

Randomised controlled trials have explored the impact of mobile apps on self-care outcomes in HF patients. For example, the ManageHF4Life app, combined with a Fitbit activity monitor and scale, showed a reduction in symptom burden at 6 weeks compared to usual care in recently discharged patients [52], although this effect was not sustained. The HeartMapp app demonstrated improvements in self-care and patient knowledge but did not impact quality of life or depression levels [53]. Gamification, as seen in cardiac rehabilitation programs, also shows promise in enhancing patient motivation and adherence to exercise [54].

A review of HF-related social media content [55] revealed a significant amount of educational material for patients and clinicians, as well as research promotion. However, significant challenges remain in the use of digital solutions for patient education, including potential inequities in access based on socio-economic status and digital literacy, the reliability of physiological data from monitoring tools and the algorithms used to guide diagnosis and treatment, the potential strain on healthcare resources from the vast inflow of clinical data and ensuring regulatory compliance (including data protection). Further research is warranted to demonstrate the benefits of digital solutions in patient education before their widespread integration into existing care pathways and electronic health records.

## Discussion

Digital solutions offer significant potential to enhance access to information and personalise learning experiences for both patients and clinicians in heart failure management, fostering more equitable knowledge sharing. For patients, these tools can improve self-care practices, medication adherence, and overall health behaviors. Meta-analyses have indeed demonstrated that various health education technologies are associated with a considerable reduction in hospitalisation rates and HF admissions [56, 57]. A large meta-analysis of 28 RCTs investigating smartphone apps for HF self-management found comparable efficacy across different app features, with reported benefits influenced by the type of technology, organisational support, feedback mechanisms, and the level of care provided to control groups [58].

The effectiveness of digital health education in improving HF outcomes is closely linked to the involvement and knowledge of clinicians in implementing these platforms. The TIM-HF2 trial, which incorporated patient education and structured monthly telephone interviews led by healthcare professionals, showed a reduction in days lost to unplanned cardiovascular hospital admissions and lower all-cause mortality compared to usual care [32]. This contrasts with the earlier TIM-HF trial, where the lack of involvement of usual clinicians in clinical decision-making resulted in no significant differences in mortality or hospitalisations [59]. Similarly, the BEAT-HF trial found no difference in readmission rates when the patient’s usual clinicians were not actively engaged, illustrating how the ‘care cycle’ breaks down when digital interventions are implemented without structured patient engagement or dedicated training for the healthcare professionals involved [60].

However, the integration of digital solutions into HF education is not without its challenges. Disparities in access to digital technologies and digital literacy among both patients and clinicians remain significant hurdles [26, 61]. Technical issues such as problems with connectivity, device compatibility and the need for ongoing maintenance and updates can also impede effective implementation. Ensuring the quality and reliability of educational content is paramount, as many digital platforms lack robust mechanisms for real-time interaction and verification [26].

The ongoing advancements in artificial intelligence and immersive technologies like virtual and augmented reality offer promising avenues for creating more personalised and engaging educational tools. Future research should prioritise well-designed randomised controlled trials to rigorously evaluate the efficacy of various digital solutions in improving both clinician knowledge and patient outcomes. Furthermore, initiatives aimed at enhancing digital literacy and ensuring equitable access to these technologies are crucial for the development of uniform guidelines and quality assurance frameworks. Collaboration among healthcare institutions, technology developers and policymakers is essential to maximise the long-term impact of digital education on the knowledge and skills of clinicians and clinical outcome in patients with HF [4, 8, 26, 44].

## Conclusion

The advent of digital solutions is fundamentally reshaping patients’ and healthcare professionals’ education and the management of heart failure, offering unprecedented opportunities for more accessible and potentially more effective training that could ultimately lead to improved outcomes, reduced healthcare cost and improve patients’ and healthcare professionals’ satisfaction. From well-established virtual platforms to the more futuristic realm of virtual reality educational tools, the landscape of HF education for patients and healthcare providers is undergoing rapid transformation.

However, realising the full potential of digital solutions in HF education requires a concerted effort from healthcare professionals, patients, and policymakers. Healthcare professionals must be willing to adopt these technologies and integrate them thoughtfully into clinical workflows, supported by appropriate training to ensure effective and safe use. Policymakers, in turn, must establish a regulatory environment that encourages innovation while maintaining rigorous standards for patient safety and data protection. Equally essential is the development of sustainable financial models that can support not only the implementation of digital educational tools but also the broader HF care cycle. Without appropriate reimbursement frameworks and funding mechanisms, even the most promising digital solutions may fail to achieve meaningful, long-term impact.

Furthermore, it is imperative to actively address the digital divide, ensuring equitable access to these technologies for all patients and healthcare professionals, irrespective of socioeconomic status or geographical location. This may require strategic investments in digital infrastructure as well as subsidies for low-income individuals. Importantly, digital solutions should also be leveraged to enhance shared decision-making, enabling patients to engage meaningfully with their care by providing clear, accessible, and evidence-based information. Such tools have the potential to empower patients to participate more actively in treatment choices and to align management strategies with their values and preferences. Finally, a sustained commitment to rigorous research and development is essential to refine and expand the capabilities of digital solutions in HF education and to ensure that their implementation leads to measurable and equitable improvements in clinical care.

In summary, digital solutions hold significant promise for revolutionising HF education both for patients and HCPs. By proactively addressing the existing challenges and thoughtfully leveraging technological advancements, healthcare professionals can enhance the education and support they provide to both their colleagues and their patients, ultimately leading to improved management and better outcomes for individuals living with heart failure.

## Key References

- Bozkurt B, Ahmad T, Alexander K, et al. HF STATS 2024: Heart Failure Epidemiology and Outcomes Statistics—An Updated 2024 Report from the Heart Failure Society of America. J Card Fail. 2025;31(1):66–116.
  - ○ This is the most comprehensive and up-to-date source on global heart failure epidemiology, outcomes, and temporal trends. It provides the strongest contemporary justification for scalable digital solutions in HF education and care delivery.
- Shahim B, Kapelios CJ, Savarese G, Lund LH. Global public health burden of heart failure: An updated review. Card Fail Rev. 2023;9:e11.
  - ○ This review synthesises worldwide HF prevalence, incidence, and prognostic trends, reinforcing the urgency of innovative educational and digital strategies to manage rising healthcare demand.
- Stevenson LW, Ross HJ, Rathman LD, Boehmer JP. Remote monitoring for heart failure management at home. *J Am Coll Cardiol*. 2023;81(23):2272–2291. doi:10.1016/j.jacc.2023.04.010. PMID:37286258.
  - ○ This landmark analysis demonstrates that remote monitoring improves outcomes only when integrated into a structured care cycle with timely, appropriate clinical responses—supporting the concept of closed-loop digital care.
- Koehler F, Koehler K, Deckwart O, et al. Efficacy of telemedical interventional management in patients with heart failure (TIM-HF2). Lancet. 2018;392(10152):1047–1057.
  - ○ One of the most definitive telemedicine trials in HF, proving that digital interventions reduce hospitalisation days and mortality when embedded within clinician-led, protocol-driven care systems.
- Wagenaar KP, Broekhuizen BDL, Jaarsma T, et al. Effectiveness of the ESC/HFA website “heartfailurematters.org” in patients with stable heart failure: The e-Vita HF trial. Eur J Heart Fail. 2019;21(2):238–246.
  - ○ A key randomised trial demonstrating that structured digital patient education improves self-care and short-term quality of life in HF.
- Cook DA, Hatala R, Brydges R, et al. Technology-enhanced simulation for health professions education: A systematic review and meta-analysis. JAMA. 2011;306(9):978–988.
  - ○ A foundational meta-analysis establishing that digital simulation improves clinician knowledge, skills, and behaviour, forming the educational evidence base for digital HF clinician training.

## Data Availability

No datasets were generated or analysed during the current study.
